# Acculturation and self-reported health among Hispanics using a socio-behavioral model: the North Texas Healthy Heart Study

**DOI:** 10.1186/1471-2458-10-53

**Published:** 2010-02-02

**Authors:** Katandria L Johnson, Joan F Carroll, Kimberly G Fulda, Kathryn Cardarelli, Roberto Cardarelli

**Affiliations:** 1 Primary Care Research Institute, University of North Texas Health Science Center at Fort Worth, 855 Montgomery Street, Fort Worth, TX, USA; 2 Department of Integrative Physiology, Graduate School of Biomedical Sciences, University of North Texas Health Science Center at Fort Worth, 3500 Camp Bowie Blvd Fort Worth, TX, USA; 3 Department of Family Medicine, Texas College of Osteopathic Medicine, University of North Texas Health Science Center at Fort Worth, 855 Montgomery Street, Fort Worth, TX, USA; 4 Center for Community Health, University of North Texas Health Science Center at Fort Worth, 3500 Camp Bowie Blvd. Fort Worth, TX, USA; 5 Department of Epidemiology, School of Public Health, University of North Texas Health Science Center at Fort Worth, USA

## Abstract

**Background:**

Acculturation is a continuous, firsthand contact with other cultures functioning at both group and individual levels and is reflected in our culturally diverse society, calling for a greater understanding of the environmental and cultural impact on health. Self-reported health (SRH), a robust and well validated predictor of future mortality for all racial/ethnic groups, has been differentially reported by Hispanics compared to whites, especially based on their acculturation status. This study investigated the relationship between acculturation and SRH among Hispanics. An adapted Andersen framework was used to develop logistic regression models to assess for an association between acculturation and general health status.

**Methods:**

Hispanic participants (n = 135), as part of the North Texas Healthy Heart Study, were administered standardized questionnaires on acculturation, psychosocial measures which included sense of control, stress, depression and social support and a single item SRH measure. In addition, physiological measurements and demographic characteristics including age, gender, body mass index, medical history, and socioeconomic status were also obtained.

**Results:**

Bivariate analyses found Mexican-oriented participants 3.16 times more likely to report fair/poor SRH compared to Anglo-oriented Hispanics. Acculturation was also associated with SRH in multiple regression models controlling for enabling, need, and predisposing factors together (OR: 3.53, 95% CI: 1.04, 11.97).

**Conclusions:**

Acculturation status was associated with SRH after accounting for other underlying factors. Medical and public health professionals should promote the use of acculturation measures in order to better understand its role in Hispanic behaviors, health outcomes and health care use. Such research findings will contribute to the design of culturally sensitive prevention and treatment strategies for diverse and immigrant populations.

## Background

Acculturation is defined as continuous, firsthand contact with other cultures functioning at both group and individual levels [1]. Such contact is an iterative process by which an individual assimilates with the sociolinguistic and cultural norms of their host country, thereby facilitating access to certain products and services, such as health care. Acculturation is reflected in our culturally diverse society, calling for a greater understanding of the environmental and cultural impact on health [1].

Higher levels of acculturation have been associated with various health outcomes, including mortality [2-4], adverse health behaviors [5], mental health disorders [6] and heart disease status, such as hypertension [7,8]. For example, hypertension rates have been associated with the acculturation continuum in middle aged Mexican Americans [8]. After controlling for potential predisposing (age, sex, marital status), enabling (insurance, employment), and need (number of chronic diseases, physical symptom scale, self-rated health, worry about health) factors, Mexican Americans who were more acculturated to U.S. norms had lower hypertension rates than the less acculturated subjects [8]. Thus, the link between acculturation and disease status may be explained by one's ability to identify and understand their health status within the context of a host country whose socio-cultural norms have not been assimilated to that individual. The role of acculturation regarding the mental health status of Hispanics with family support can function as a moderator or mediator and is differential across immigration status [9,10]. Such research findings support the importance of including psychosocial risk factors and length of stay in the host country when examining acculturation's influence on health.

Self-reported health (SRH), a robust and well validated predictor of future mortality for all racial/ethnic groups, has been differentially reported by Hispanics compared to whites, especially based on their acculturation status [11-13]. A study by Shetterly et al. [14] revealed that Hispanics were 3.6 times more likely to report fair or poor health compared to whites, with acculturation being the strongest explanatory factor. Another study using data from the National Health Interview Survey (NHIS) from 1989-1994 found poor SRH as a weak predictor of subsequent mortality risk among less acculturated individuals [15]. However, the association between poor SRH and mortality risk increased with U.S. acculturation, with differential effects noted across language, place of birth, and length of stay in the U.S. [15]. Cultural practices and linguistic competency of the individual across a span of time in a host country can moderate or exacerbate access to healthcare providers and the ability to secure insurance. Furthermore, the role of acculturation, specifically one's level of assimilation to the health behaviors of the host country, may affect how health is perceived.

Although studies have assessed how acculturation status predicts the status of various health outcomes, it remains unclear whether acculturation status truly predicts one's SRH after taking into account socioeconomic measures, psychosocial factors, health behaviors, chronic disease status, and access to care measures. Moreover, the investigators propose that these factors can be more clearly assessed using an adapted framework developed by Andersen [16]. We hypothesized that lower levels of U.S. acculturation are associated with poor/fair SRH status and this relationship is confounded by predisposing, enabling, and need factors related to the Andersen model discussed below.

## Methods

### Andersen socio-behavioral model

**Figure 1:**
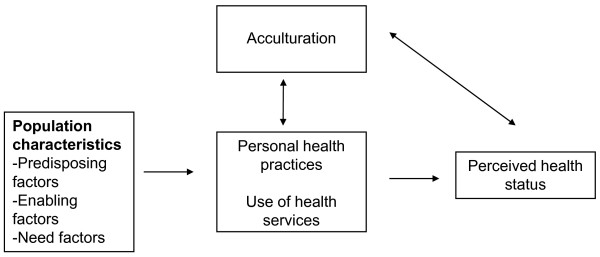
**Study framework adapted from the Andersen Socio-behavioral Model (Andersen 1995)**.

Several iterations of the Andersen model have been developed to better understand how health services use, and ultimately health status, is impacted by social and behavioral factors [16]. The original model was developed in the 1960's to help understand health service use patterns at the individual level in the context of factors that may facilitate or impede use and the need for care. The present study applied an adapted model to examine how acculturation's association with health status is impacted by three groups of factors suggested by Andersen, including predisposing factors (demographic characteristics and education level), enabling factors (income and health insurance status, and having a healthcare provider), and need factors (disease status [hypertension, diabetes mellitus and hyperlipidemia], body-mass-index [BMI], and psychosocial variables) (Figure 1). Each group of factors is also referred to as determinants.

The model allows a causal ordering and explanation with predisposing factors functioning as external attributes that are non-modifiable, enabling factors functioning as individual resources, and underlying need factors driving one to seek health care and change health behaviors. Each group of factors may be conceived as making an independent contribution to predicting health care use and health status. This approach was taken since acculturation's influence on health status is multi-factorial, affected by personal circumstances, socioeconomic measures, and disease status. This model also allows a systematic approach to assess the relationship between acculturation and health status and the impact of each determinant. These determinants of health status are theoretically mediated through personal health practices and use of health services. However, due to available data, the present study will only assess the relationships among acculturation, the determinants, and health status.

### Participant selection and protocol administration

The Dallas- Fort Worth (DFW) metropolitan area is a major national hub for migration from various parts of the world and its largest minority population is of Hispanic origin. Close to 8.4 million Hispanics live in Texas, with an estimated 7 million being of Mexican descent [17]. Research participants were recruited from the North Texas Healthy Heart (NTHH) study, a cross-sectional study involving a convenience sample of 371 non-Hispanic whites, non-Hispanic blacks, and Hispanics/Latinos. One-hundred and thirty-five Hispanic/Latinos were recruited from 12 participating sites of the North Texas Primary Care Practice-Based Research Network (NorTex). NorTex is a collaborative network of community organizations and primary care clinics serving low-income, under-represented populations of the DFW metropolitan area. The 12 sites that participated in the NTHH study included four academic community-based clinics, three county community health centers, four solo-practitioner private practices, and one federally qualified health center.

All participants were interviewed in the language of their preference, i.e. Spanish or English. All written portions of the research protocol were translated into Spanish.

### Inclusion/Exclusion Criteria

In the NTHH study, all subjects were 45 years of age or older and self-identified as non-Hispanic white, non-Hispanic African American, or Hispanic. Exclusion criteria included a history of cardiovascular disease (coronary artery disease, peripheral arterial disease, history of myocardial infarction or stroke, or congestive heart failure), renal failure, or liver failure. Subjects were screened for eligibility over the phone and in person. Study procedures occurred at the University of North Texas Health Science Center at Fort Worth, Primary Care Research Institute. A total of 718 potential subjects were screened. Of those screened, 599 were eligible to participate, and 447 were invited to participate. These 447 eligible participants were invited consecutively until each racial/ethnic group had met preset recruitment goals. The remaining eligible participants were included on a waitlist. Three hundred seventy-one participants entered the study, representing an 83% recruitment rate. A total of 135 Hispanics completed the NTHH study. These Hispanic subjects were included in the current analysis. Study procedures were approved by the University of North Texas Health Science Center and John Peter Smith Hospital Institutional Review Boards, and informed consent was obtained from all invited participants.

### Dependent Variable: Self-Reported Health (SRH) Status

Health status was ascertained using a self-reported single-item indicator that has been shown to be a reliable predictor of future population mortality [11]. Responses from the SRH question, "In general, how would you rate your health?" were categorized as "excellent/very good/good" versus "fair/poor" [11].

### Independent Variable: Acculturation

Acculturation was measured using the Acculturation Rating Scale for Mexican Americans (ARSMA)-II, which is a commonly used scale to assess acculturation processes through an orthogonal, multidimensional approach by measuring cultural orientation toward the Mexican culture and the Anglo culture [18,19]. Before administering the subscales, questions regarding demographic information, birth country, and years of residence in the U.S. were elicited.

The ARSMA-II is comprised of a set of two subscales, the Anglo Orientation Subscale (AOS) (Cronbach's α = 0.83, μ = 3.82, σ = 0.57) [18] and the Mexican Orientation Subscale (MOS) (Cronbach's α = 0.88; μ = 3.28, σ = 0.84) [18], which were used to categorize participants. Responses to questions were "not at all," "very little or not very often," "moderately," "most or very often," or "extremely or almost always." The AOS included 30 contextual questions regarding how often and to whom participants spoke English, e.g. "My friends are of Anglo origin." The MOS included 18 contextual questions regarding how often and to whom participants spoke Spanish, e.g. "I enjoy reading books in Spanish." According to ARSMA-II specifications, those with low acculturation scores (≤ -0.07) were categorized as Mexican-oriented and those with high acculturation scores (>-0.07) were categorized as Anglo-oriented.

### Covariates

The covariates were organized and analyzed as groups of variables with each cluster representing a determinant of health status (i.e., predisposing factors, enabling factors, and need factors) [16,20].

### Predisposing factors

The NTHH study utilized standardized questions from the Behavioral Risk Factor Surveillance System to collect demographic information. Age was measured as a continuous variable (years), and gender was dichotomized as male or female. Education was measured by the question, "What is the highest grade or year of school that you completed?" Responses were then categorized as "less than high school", "high school graduate/GED", or "some college or greater".

### Enabling factors

Income was categorized as "below $20,000/year" and "$20,000/year or greater". Responses to having health insurance and a personal healthcare provider were both dichotomized as "yes" or "no". Participants with Medicare or Medicaid were categorized as having health insurance.

### Need factors

Medical histories were collected as need factors for this study. The presence of hyperlipidemia, hypertension, and diabetes mellitus status were ascertained for each participant. Hyperlipidemia was considered to be present if the participant had a low density lipoprotein (LDL) level = 160 mg/dL, reported being previously diagnosed with high cholesterol, or was taking a lipid lowering medication. Hypertension was considered present if the participant's blood pressure reading was greater than or equal to 140 mmHg for systolic or 90 mmHg for diastolic pressures. Blood pressure was measured using standard procedures. Two blood pressures from the participant's non-dominant arm were averaged after a 5-minute rest period in a sitting position. Hypertension was also considered if the subject reported a previous diagnosis of hypertension, or if the subject was taking antihypertensive medications.

Diabetes was considered present if the fasting glucose level was greater than or equal to 126 mg/dL, if the subject reported being previously diagnosed with diabetes, or if the subject was taking any anti-diabetic medication. Weight, height, and blood pressure (millimeters of mercury [mmHg]) were also measured. Height was measured to the nearest 0.25 inch, and weight was measured to the nearest 0.25 lb using a standard balance scale. Height and weight measurements were used to calculate body-mass index for each subject using the Quetelet's equation (kg/m^2^).

### Psychosocial measures

The Perceived Stress Scale (PSS), a 10 item, self-administered questionnaire with scores ranging from 0 to 33, measured the degree to which participants' life events were considered stressful (Coefficient alpha reliability scores = 0.84, 0.85, and 0.86 for the three original study samples) [21] with no differences across gender found (p < 0.01) [21]. The Sense of Control Scale (SOC) [22] is an eight item survey (score range: -0.2 to 2.0) used to measure participants' control over personal outcomes and how it relates to their ability to achieve a desirable or undesirable outcome (alpha = 0.68, μ = 0.68) [22].

The Social Support Scale is a 4 item survey (score range: 0 to 4) used to measure participants' self-reported emotional and instrumental support (alpha reliability = 0.88, μ = 0.85) [23]. The Center for Epidemiological Disease Studies (CES-D) Scale is a 20 item survey (score range: 0 to 49) used to identify depressive symptomatology (reliability alpha = 0.85) [24].

### Statistical Analysis

All analyses were conducted using SPSS version 15.0 software [25]. Descriptive statistics, crude logistic regression and multiple logistic regression analyses were performed to assess whether acculturation was associated with SRH status. Counts and frequencies were calculated for categorical data, and means and standard deviations were calculated for continuous variables. An analysis of Mexican-oriented and Anglo-oriented Hispanics across several demographic, financial, and medical characteristics was conducted using the student t-test for continuous variables and chi-square analyses for categorical variables (Table 1).

**Table 1:** Study population characteristics (North Texas Healthy Heart Study, N = 135)

Characteristics	Mexican-oriented (n = 48)	Anglo-oriented (n = 81)	p-value*	Total
**Self-Reported Health**(%)			<0.01	
*Excellent/Very Good/Good*	53.2	78.3		62.6
*Fair/Poor*	46.8	21.7		37.4

**Age **mean (Standard deviation)	53.65 (7.2)	57.7 (8.0)	<0.01	55.1 (7.8)

**Gender**(%)			0.36	
*Female*	63.0	70.8		65.9
*Male*	37.0	29.2		34.1

**Education **(%)			<0.01	
*Less than High School*	65.4	27.1		51.2
*High School or Greater*	34.6	72.9		48.8

**Income **(%)			0.05	
*Below $20,000*	45.9	27.9		39.3
*$20,000 or Greater*	54.1	72.1		60.7

**Psychosocial Factors, mean (SD)**				
*Stress Score*	14.3 (6.4)	14.4 (7.0)	0.94	14.4 (6.6)
*Sense of Control Score*	0.6 (0.5)	0.7 (0.5)	0.12	0.6 (0.5)
*Social Support Score*	1.8 (0.5)	1.7 (0.6)	0.61	1.8 (0.5)
*Depression Score*	13.7(10.5)	12.2 (10.3)	0.41	13.2 (10.4)

**BMI**, mean (SD)	31.1 (5.9)	32.1 (6.2)	0.40	31.5 (6.0)

**Medical History **(%)				
*High Cholesterol*			0.43	
Yes	24.7	18.8		77.5
No	75.3	81.3		22.5
*Hypertension*			0.05	
Yes	40.7	58.3		47.3
No	59.3	41.7		52.7
*Diabetes Mellitus*			0.53	
Yes	22.2	27.1		24.0
No	77.8	72.9		76.0

**Healthcare Provider **(%)			<0.01	
*Yes*	62.8	93.6		74.4
*No*	37.2	6.4		25.6

**Health insurance **(%)			<0.01	
*Yes*	48.8	87.5		63.3
*No*	51.3	12.5		36.7

*Two-tailed tests

To address the main research questions, a series of logistic regression models were developed to assess whether the relationships between the primary independent variable and outcome were confounded by predisposing factors (model 1), enabling factors (model 2), or need factors (model 3) based on the Andersen socio-behavioral model (Table 2).

**Table 2:** Bivariate and Multiple Logistic Regression Analyses of Fair/Poor Health Status (North Texas Healthy Heart Study, N = 135)


	**Bivariate**		**Model 1**		**Model 2**		**Model 3**		**Model 4**	

	**OR**^**a**^	**95% CI**^**b**^	**OR**	**95% CI**	**OR**	**95% CI**	**OR**	**95% CI**	**OR**	**95% CI**

Acculturation										
Mexican-oriented	3.16	1.37, 7.25	1.87	0.73, 4.81	3.65	1.35, 9.89	3.67	1.35, 9.98	3.53	1.04, 11.97

**Predisposing Factors**										
Age^c^	1.00	0.95, 1.04	0.98	0.93, 1.03					0.98	0.91, 1.05
Gender										
Male	0.95	0.44, 2.02	1.37	0.58, 3.24						
Education										
Less than High School	4.16	1.93, 8.97	4.06	1.64, 10.05					2.42	0.75, 7.77

**Enabling Factors**										
Income										
Below $20,000	1.82	0.84, 3.93			1.45	0.60, 3.47			1.14	0.39, 3.28
No health Insurance	1.39	0.67, 2.88			0.74	0.29, 1.88			0.35	0.11, 1.13
No healthcare provider	1.69	0.73, 3.90			1.13	0.42, 3.04			1.91	0.61, 5.91

**Need Factors**										
Diabetes Mellitus present	2.10	0.92, 4.76					0.98	0.32, 2.99		
Hypertension present	1.61	0.78, 3.29					1.54	0.62, 3.84	1.68	0.62, 4.58
Hyperlipidemia present	2.26	0.94, 5.42					1.91	0.62, 5.88		
Body Mass index^c^	1.06	0.99, 1.12					1.07	0.99, 1.17		
Perceived stress^c^	1.07	1.01, 1.14					1.03	0.95, 1.12	1.03	0.94, 1.13
Sense of control^c^	0.26	0.12, 0.58					0.43	0.17, 1.07	0.46	0.16, 1.31
Social support^c^	0.97	0.52, 1.82					0.59	0.25, 1.37		
Depression symptomatology^c^	1.08	1.04, 1.12					1.07	1.01, 1.13	1.06	1.01, 1.32

*Note*: ^a^Odds Ratio; ^b^Confidence Interval; ^c^Assessed as a continuous variable

The full multiple logistic regression model, which assessed the influence of all three groups of factors, was analyzed for multi-collinearity and selected potential interactions. Only variables that were statistically related to either the primary independent variable (acculturation) or the dependent variable (SRH) were included in the final model. Thus, the final model did not include all variables. Variables excluded were gender, diabetes, hyperlipidemia, BMI, and social support. No collinear relationships or interactions were identified. Statistical significance was measured at the alpha 0.05 level.

Data were missing for 0.83% of the total responses for items composing acculturation, resulting in 2.1% missing scores. Missing data were imputed for responses to acculturation using the individual mean imputation method, which imputed a value based on how a subject responded to other questions in the scale. This method was chosen because of its simplicity and accuracy [26]. Missing data were not imputed for income, education, stress, depression, BMI, high blood pressure, diabetes, healthcare provider, and insurance, as the rates were less than 1%. There were no missing data for sense of control or cholesterol. This resulted in final sample sizes of 123 for model 1, 107 for model 2, 121 for model 3, and 105 for model 4.

## Results

Descriptive characteristics of the NTHH participants are presented in Table 1. Although it is not shown in the table, 98.4% of the participants were of Mexican-origin. More than half of all Hispanics reported excellent/very good/good health, but Mexican-oriented Hispanics rated their health lower than Anglo-oriented Hispanics. Mexican-oriented Hispanics were more likely to report less than a high school education than Anglo-oriented participants or income below $20,000. The majority of Mexican-oriented participants reported access to a primary care yet had no health insurance.

Logistic regression results are found in Table 2. Bivariate regression analyses revealed Mexican-oriented Hispanics were over 3 times more likely to report fair/poor health compared to Anglo-oriented Hispanics. Other statistically significant predictors included education, sense of control, stress, and depression scores in that participants with education levels less than high school were 4.16 times more likely to report fair/poor self-health ratings compared to those who had higher levels of education. In addition, Hispanics who reported high levels of stress or depression were 1.07 and 1.08, respectively, times more likely to report fair/poor health. Conversely, Hispanics who reported a higher sense of control were less likely to report fair/poor health.

Multiple logistic regression results can also be found in Table 2. In model 1, acculturation was not significantly associated with poor/fair health once predisposing factors were included in the model. High education status (i.e., high school or greater) remained a protective factor from fair/poor health status. In models 2 and 3, Mexican-oriented acculturation remained significantly associated with fair/poor health and was not impacted by enabling or need factors, respectively. Model 4 included only variables that were statistically significantly related to the independent or dependent variable within the bivariate and multivariate models. This final model found acculturation to be significantly associated with fair/poor health status. Depression symptomatology was the only other variable that remained significantly associated with fair/poor health in all models.

## Discussion

The study's aim was to better understand the relationship between acculturation and SRH. The importance of this research results from conflicting evidence in the literature and perceptions of Hispanic health. Many health disparity researchers view Hispanics as a "healthy" but economically-disadvantaged population and have used terms such as the "Hispanic paradox" [27]. However, it remains unrecognized that the mental and physical suffering and morbidity is as important as mortality outcomes and surrogate disease measures. Moreover, extensive research has demonstrated that acculturation can have both negative and protective effects depending on the outcome of interest, such as certain health behaviors or health care use [28]. Therefore, SRH was chosen as the outcome variable of interest in the present study since it conceptually functions as a composite measure of mental and physical well being. In addition, this form of health status reporting has been shown to be predictive of adverse health outcomes, including overall mortality for all racial/ethnic groups [13].

The Andersen socio-behavioral approach taken by this study allowed a better understanding of how acculturation impacts health status by systematically assessing clusters of determinant factors and their impact on SRH. Using this approach, Mexican-oriented acculturation remained an independent predictor of fair/poor health in the final model. This finding supports the notion that self-rated health may be affected by a person's level of acculturation while controlling for certain predisposing, enabling, and need factors. Although data are not shown, the authors conducted multiple logistic regression analyses for each predisposing variable with acculturation and only education was found to function as a confounder.

Previous research has argued that acculturation studies have often overlooked the importance of socioeconomic status to better understand the relationship between acculturation and health outcomes [29]. In fact, this study's results supported the theory that acculturation functions in a greater social context, and not only at the individual level, with education being one of the important variables to consider. Our results were similar to that of Markides and Martin [30] who suggested lower SRH of Mexican Americans in Texas was solely due to education and income. Education may have improved health by increasing effective agency and physical functioning [22] and enhanced a sense of control that enabled one to select a healthy lifestyle [31]. Other studies have reported associations between lower SRH with lower education and income [32-37]. The variations among studies assessing the relationship between acculturation and outcomes, such as health behaviors and disease markers, also demonstrated differences on how education impacts these associations. Education functioned as a confounder in some studies while not in others. There are many possible explanations for these inter-study variations, including geographic variation, differences among Hispanic subgroups or other races/ethnicities, and how acculturation was measured [29,38,39]. Many studies utilized proxy measures of acculturation, such as the number of years residing in the United States or primary language spoken. However, it has been suggested that these measures assumed stereotypes and were not accurate measures of acculturation [29].

Although not measured in the current study, the association between acculturation, determinants of health status, and health status is conceptually thought to be mediated by health care use and health behaviors [16]. However, conflicting evidence exists in the literature since most studies related to health behaviors have found Anglo-oriented acculturation to be associated with higher rates of poor behaviors such as smoking and obesity [36]. Conversely, Mexican-oriented acculturation has been associated with lower health care use and health status. One possible explanation is increased reporting of lower SRH among Mexican-oriented Hispanics. One study found Hispanics with a lower education status tended to make more extreme choices when responding to questions in surveys compared to those with higher education levels [40]. Also, Hispanics might have been less willing to rate their own health highly because it was not a commonly accepted practice among older, more traditional Hispanics [14]. Another potential explanation is that Hispanics tended to somatize their mental and emotional health into physical health leading to lower ratings of health [41].

There are several strengths and limitations to the study that are worth noting. Although statistically significant results were found, the sample size was relatively small. Also, caution is advised when generalizing the results to other geographic regions since variation may exist. Hispanics were discussed as a homogenous group when, in fact, there may be significant variations among Hispanic sub-populations. The strengths of this study include the use of a validated instrument to measure acculturation and psychosocial measures, which past literature may not have addressed. However, the subjective nature of surveys must be taken into perspective. As mentioned previously, response to study questions may be influenced by one's educational level or other related factors and consequently skew study results. Finally, the cross-sectional nature of this study prohibits any assumptions of causality.

## Conclusions

Larger statistical samples are needed to examine how psychosocial variables and acculturative factors may contribute to the causal pathways of various disease outcomes. In addition, more research that promotes the use and improved definition of acculturation measures as it relate to theoretical models of public health will increase knowledge of the role of acculturation in Hispanic health behaviors, health outcomes, and health care use [28]. Such research findings will contribute to the design of culturally sensitive prevention and treatment strategies for racially/ethnically diverse and immigrant populations.

## Competing interests

The authors declare that they have no competing interests.

## Authors' contributions

KLJ carried out data collection, entry, statistical analysis and manuscript development. JFC helped conceive the study as co-investigator and reviewed the manuscript. KGF participated in statistical data management, analysis, manuscript development, review, and submission. KC participated in the study's conception and design and manuscript review. RC was the primary investigator, helped conceive the study, conducted data analysis, helped draft and review the manuscript. All authors read and approved the final manuscript.

## Author's Information

Katandria Johnson, MA, MS, CCC-SLP is a DrPH social and behavioral sciences of public health graduate student, linguist, and trilingual speech-language pathologist. She also works as a research associate for the Primary Care Research Institute at the University of North Texas Health Science Center at Fort Worth. Research interests include international health and health disparities among pediatric populations with special healthcare needs.

Joan F. Carroll, Ph.D., F.A.C.S.M. is an Assistant Professor in the Department of Integrative Physiology at the University of North Texas Health Science Center. Dr. Carroll's research interests include cardiovascular function in obesity, using both human and animal models, and ethnic disparities in the relationship between body composition and inflammation.

Kimberly Fulda, DrPH is an Assistant Professor in the Department of Family Medicine and the Associate Director of the Primary Care Research Institute at the University of North Texas Health Science Center. Dr. Fulda's specialty is biostatistics and research design. Her research interests include children's health, maternal and child health, as well as public health.

Kathyrn Cardarelli, PhD is an Assistant Professor in the Department of Epidemiology and Director of the Center for Community Health at the University of North Texas Health Science Center. Dr. Cardarelli's research interests include the influence of social and psychosocial factors (including socioeconomic position, discrimination, sense of personal control, and social support) on chronic disease and pregnancy outcomes and mechanisms involved in these relationships; community-based research; and policy and methods related to population health and health disparities

Roberto Cardarelli, DO, MPH is an Associate Professor in the Department of Family Medicine and the Executive Director of the Primary Care Research Institute at the University of North Texas Health Science Center. Dr. Cardarelli's research interests include improving primary care practice, physicians disciplined by state medical boards, chronic disease management, evidence-based reviews, and health disparities in point-of-care practice. His main interest in medical care is chronic disease management.

## Pre-publication history

The pre-publication history for this paper can be accessed here:

http://www.biomedcentral.com/1471-2458/10/53/prepub
